# Maintaining IV Patency Throughout Pharmacokinetic Studies Through a Needle‐Free Blood Draw Device

**DOI:** 10.1002/prp2.70333

**Published:** 2026-10-16

**Authors:** William Naviaux, David Leinweber, Heather Barkholtz

**Affiliations:** ^1^ Pharmaceutical Sciences, School of Pharmacy University of Wisconsin‐Madison Madison Wisconsin USA; ^2^ Forensic Toxicology, Environmental Health Sciences Wisconsin State Laboratory of Hygiene Madison Wisconsin USA; ^3^ Department of Family Medicine and Community Health, School of Medicine and Public Health University of Wisconsin‐Madison Madison Wisconsin USA

## Abstract

Pharmacokinetic (PK) studies require temporally precise measurements of drug concentrations in biological matrices, most commonly whole blood or plasma. However, repeated venous sampling may increase participant burden and complicate PK‐intensive study designs. Needle‐free blood collection devices that access peripheral intravenous (IV) catheters may provide an alternative approach to repeated venipuncture while maintaining sampling schedules and specimen quality. This pilot study evaluated the feasibility of integrating the PIVO Pro needle‐free blood collection device into a PK‐intensive human laboratory study of inhaled Δ^8^‐tetrahydrocannabinol (Δ^8^‐THC), Δ^9^‐THC, and placebo. Participants completed a screening visit and three dosing visits conducted in a university research facility equipped with a full‐scale driving simulator. During each dosing visit, venous blood samples were collected at nine time points over approximately 8 h through an indwelling peripheral IV catheter using the PIVO device. All participants reported that study procedures, including blood collection, were acceptable. Approximately 40% of vascular access sites required replacement, most commonly within the first hour and typically associated with poor initial blood return. Despite slower flow compared with standard venipuncture, sampling was generally sufficient to maintain the PK sampling schedule. One mild adverse event related to vascular access (localized bruising) was observed. These findings demonstrate the operational feasibility of needle‐free blood collection through indwelling IV catheters in a PK‐intensive research protocol conducted outside a traditional clinical research unit. Further studies are warranted to compare analytical performance and access durability relative to conventional venous sampling approaches.

## 1 Background

Pharmacokinetic (PK) studies require accurate and temporally precise measurement of drug concentrations in biological matrices, with whole blood and plasma remaining the gold standard for most clinical applications [1, 2, 3]. However, repeated venous blood sampling increases participant burden and may limit the feasibility of PK‐intensive study designs. Minimizing invasiveness while maintaining sample integrity is therefore a key operational consideration in such studies.

Standard phlebotomy can be time‐consuming and may require repeated venipunctures, which can compromise sampling schedules, disrupt protocol workflows, increase participant burden, and increase risks of adverse events [4, 5]. Peripheral intravenous (IV) catheters are commonly used; however, they present distinct technical limitations. In particular, blood obtained through standard IV access may be susceptible to hemolysis, limiting suitability for plasma‐based analysis [6]. Maintaining catheter patency over repeated draw attempts is also challenging, as IV systems are designed primarily for infusion rather than withdrawal, and repeated aspiration rapidly degrades catheter patency.

Alternative approaches to repeated venous blood collection are therefore of interest, particularly in non‐clinical research settings. The PIVO Pro needle‐free blood collection device (BD, Franklin Lakes, NJ, USA) enables blood collection through existing peripheral IV catheters without direct needle access [7]. Prior studies have reported favorable tolerability and reduced complication rates compared with standard IV blood draws and repeated phlebotomy in inpatient settings [8, 9]. On this basis, the PIVO Pro device was evaluated as a blood collection strategy within a PK‐intensive pilot study prioritizing both participant experience and sample quality.

## 2 Materials and Methods

### 2.1 Overall Study Design

This phase 1 study evaluated the feasibility of repeated venous blood collection during administration of inhaled ∆^8^‐tetrahydrocannabinol (∆^8^‐THC), ∆^9^‐THC, and a placebo. This study was approved by the University of Wisconsin‐Madison's Health Sciences IRB (# 2022–1124), administration of the study drugs was approved under an investigator‐initiated FDA IND (# 164750) held by the corresponding author, and the clinical trial was posted to Clinical Trials.gov (# NCT06236815).

Each participant (*N* = 5) completed four visits: a screening visit and three dosing visits. During dosing visits, participants underwent serial cognitive, psychomotor, and driving simulation assessments concurrent with intensive blood sampling to support PK analysis. Because study procedures included use of a full‐scale driving simulator, dosing visits were conducted in a university research facility rather than a clinical research unit. Medical oversight was maintained throughout all study visits, including on‐site clinical staff and supervision by a study physician.

### 2.2 Blood Collection

Venous access was established using a 20‐gauge, 1.25‐in. Nexiva closed IV catheter system with NearPort IV access (BD, Franklin Lanes, NJ, USA). A single clinician placed and maintained all IV catheters for all visits. Catheter placement was confirmed with a saline flush and secured with a standard sterile dressing. The IV catheter was intended to remain in place for the duration of the approximately 8‐h dosing visit to support repeated venous sampling without additional venipuncture. Venous blood samples were collected at pre‐dose, immediately post‐dose, every 30 min for 2 h, and at 3‐, 4‐, and 6.5‐h post‐dose (9 collections per visit).

At each collection time point, the catheter was flushed with a saline flush prior to the PIVO Pro needle‐free blood collection device (BD, Franklin Lakes, NJ, USA) insertion through the existing IV catheter. Two 10 mL tubes of blood were collected per time point. Following collection, the device was removed and the catheter was flushed with a saline flush and covered with a disinfecting cap. In cases of inadequate flow, a standardized escalation approach was implemented: device repositioning, equipment replacement, and, if necessary, establishment of new vascular access.

In addition to venous sampling, ancillary dried capillary blood spots were collected using the amasse device (OpAnd, Durham, NC, USA) at selected time points via fingerstick using single‐use lancets to puncture the pad of the finger. Capillary blood was applied to filter paper cards according to manufacturer's instructions. Capillary blood spot data are not presented in this report.

### 2.3 Participant Feedback

Participants completed a post‐study electronic survey assessing study experience and acceptability of study procedures [10, 11]. Survey items included Likert‐scale, yes/no, and free‐response questions. Items relevant to blood collection evaluated acceptability of study procedures and overall study experience.

## 3 Results

A total of 14 dosing visits were completed. One participant withdrew before completing the final dosing visit due to schedule changes. All 5 participants completed the post‐study survey. All respondents indicated that the method of data collection, including blood draws, was acceptable. Free response collects were limited; one participant described the blood collection component as “minimally unpleasant”, noting finger sticks for capillary blood microsampling as more bothersome than IV‐based blood draws.

Approximately 42% of vascular access sites required replacement during study participation. Across 14 dosing visits, 14 initial IV access sites were placed; 6 required replacement. Four of the 6 replacements occurred in a single participant. Prior works have reported failure rates of 69.2% for traditional open IV access systems [12]. Therefore, the PIVO device resulted in 42% fewer instances of IV failure than anticipated. Replacements predominantly occurred early (within the first 2 h). Early failures were associated with poor initial blood return that degraded upon subsequent draw attempts. Overall, blood flow through the device was slower than standard venipuncture but generally sufficient to maintain the sampling schedule. Flow variability was often mitigated by adjustment of catheter position, arm position, or device advancement depth.

One adverse event was recorded that is attributable to vascular access. A single participant experienced bruising and tenderness at the access site after completion of a dosing session. During this dosing visit, the participant's initial IV access site experienced poor initial blood return, as described above, and a second vascular access site was required. The first vascular access site went on to develop bruising and tenderness, which did not interfere with the participant's daily activities and resolved without intervention. No other bruising incidents were observed or reported to the study team.

## 4 Discussion

This pilot study demonstrates the operational feasibility of integrating the PIVO device into a PK‐intensive study requiring nine venous collections per visit. Prior studies have demonstrated that PIVO can provide clinically valid specimens, reduce hemolysis, and improve patient acceptability while maintaining peripheral catheter functions [8, 9, 13, 14, 15, 16]. However, these investigations were conducted primarily in inpatient clinical settings. Our findings extend this work by demonstrating that the device can also support intensive serial sampling in a non‐clinical research environment while maintaining protocol‐required collection schedules. These findings suggest that needle‐free blood collection through an existing peripheral IV catheter may be a practical approach for intensive pharmacokinetic studies where participant burden and preservation of vascular access are important considerations.

Most collection challenges occurred early in the dosing visit and appeared related to initial catheter positioning and flow limitations rather than progressive loss of access later in the session. Reductions in flow were addressed by systematically escalating intervention. First intervention was repositioning of the participant's arm. If this failed, the second intervention was obtaining a new needle‐free PIVO device and a blood collection tube was utilized to evaluate for equipment failure. Only if these interventions failed was new vascular access obtained.

Device selection was influenced by compatibility and flow considerations. A 20‐gauge catheter was selected to optimize flow characteristics while minimizing participant discomfort. Catheter securement and avoidance of sharp angulation in extension tubing were important for maintaining adequate flow. Access site distal to venous convergence points appeared to improve sampling consistency.

Most collection challenges occurred early in the study and were frequently resolved through adjustments to catheter position, arm position, or device advancement depth, suggesting that operator experience may influence successful implementation. Notably, the large multicenter implementation study by Pendleton and LaFaya excluded data from the first 20 days following implementation, citing concerns that proficiency development could confound performance outcomes [9]. Our pilot study captured all collections during initial implementation and may therefore reflect a greater influence of the learning curve. Although our study observed a higher vascular access replacement rate than has been reported in large hospital‐based evaluations of PIVO, direct comparison is challenging due to substantial differences in study design [9]. For example, a multicenter study of more than 150 000 PIVO blood collections reported a modest reduction in catheter replacement rates compared with conventional collection practices; however, hospitalized patients were monitored within routine clinical workflows, early implementation data were excluded, and catheter replacement was not required solely because blood could not be obtained through PIVO [9]. In contrast, the present study was conducted in an intensive pharmacokinetic research setting requiring repeated blood collections over an approximately 8‐h visit, where inability to obtain a blood sample necessitated replacement of venous access to maintain protocol adherence. These differences likely contributed to the higher replacement rate observed in our cohort. Additionally, four of six vascular access replacements occurred in a single participant, suggesting that individual vascular characteristics may have had a disproportionate influence on outcomes in this small pilot study.

Out of 14 total dosing visits (one participant withdrew prior to completing the final dosing visit), only 1 AE was observed related to venous access, specifically delayed bruising. Incidence rates of single venipuncture are reported as high as 10.3% in the literature [17]. By reducing the incidence of repeated venipuncture the overall number of adverse bruising incidents can be reduced. Participants all indicated that the method of data collection, including blood draws, were acceptable.

The primary cause of workplace injuries for healthcare workers is biological exposures [18]. Use of a closed IV system with needle‐free blood collection reduced sharps exposure and eliminated syringe transfers typically required for IV aspiration, potentially improving research staff safety during intensive sampling. In addition to the ethical requirements of reducing exposure to blood borne pathogens, statutory requirements under 29 CFR 1910.030 require employers to implement systems that minimize uncontrolled release of potentially infectious material, specifically calling out needleless systems [19].

## 5 Limitations

This was a small pilot study without a comparator arm using standard venipuncture or direct IV aspiration. The study was not designed to formally evaluate equivalence of analyte concentrations between collection methods. Flow characteristics and causes of catheter failure were not prospectively quantified. The standardized escalation approach to troubleshoot low blood flow was adopted over the first few dosing visits. Many initial occurrences of low blood flow resulted in the immediate establishment of new vascular access due to the study team's familiarity with direct IV aspiration. Over time, it was observed that repositioning of the participant's arm and/or the device often resolved blood flow challenges. This likely resulted in over‐estimation of vascular access site replacements. Findings should therefore be interpreted as operational observations rather than definitive performance comparisons.

## 6 Conclusion

In this PK‐intensive pilot study, integration of the PIVO needle‐free blood collection device was feasible and acceptable to participants. The approach may reduce repeated venipuncture and sharps exposure in early‐phase or non‐clinical research settings requiring frequent sampling. Controlled comparative studies are warranted to evaluate analytical equivalence and long‐term access performance relative to conventional collection methods.

## Author Contributions

**William Naviaux:** conceptualization, investigation, writing – original draft, methodology, writing – review and editing, formal analysis, data curation. **David Leinweber:** investigation, writing – review and editing, methodology, formal analysis, supervision. **Heather Barkholtz:** conceptualization, investigation, funding acquisition, writing – original draft, methodology, writing – review and editing, formal analysis, project administration, supervision, resources.

## Funding

This work was supported by Institute for Clinical and Translational Research, University of Wisconsin, Madison, National Center for Advancing Translational Sciences (Grant UL1TR002373) and Wisconsin Partnership Program (Grant 5132).

## Conflicts of Interest

The authors declare no conflicts of interest.

## Data Availability

The data that support the findings of this study are available on request from the corresponding author. The data are not publicly available due to privacy or ethical restrictions.
